# Supplementary material for: Modeling glioblastoma heterogeneity as a dynamic network of cell states
Source: Mol Syst Biol. 2021 Sep 16;17(9):e10105. doi: 10.15252/msb.202010105 (PMC8444284; doi:10.15252/msb.202010105)
Supplement: Supplementary file 6 — Source Data for Figure 5 [file MSB-17-e10105-s004.zip › Figure5A_sourcedata/GSEA_3017/nosigdb_stateD.GseaPreranked.1622795010397/NB_KI_CLUSTER5.html]

Details for gene set NB\_KI\_CLUSTER5[GSEA]

|  || Dataset | state23017 |
| Phenotype | NoPhenotypeAvailable |
| Upregulated in class | na\_neg |
| GeneSet | NB\_KI\_CLUSTER5 |
| Enrichment Score (ES) | -0.5596633 |
| Normalized Enrichment Score (NES) | -2.7488372 |
| Nominal p-value | 0.0 |
| FDR q-value | 0.0 |
| FWER p-Value | 0.0 |
Table: GSEA Results Summary

  

Fig 1: Enrichment plot: NB\_KI\_CLUSTER5      
 Profile of the Running ES Score & Positions of GeneSet Members on the Rank Ordered List

  

| PROBE | GENE SYMBOL | GENE\_TITLE | RANK IN GENE LIST | RANK METRIC SCORE | RUNNING ES | CORE ENRICHMENT || 1 | PTTG1 |  |  | 14 | 0.490 | 0.0041 | No |
| 2 | BIRC5 |  |  | 130 | 0.271 | -0.0784 | No |
| 3 | DDX11 |  |  | 211 | -0.255 | -0.1337 | No |
| 4 | TRIP13 |  |  | 294 | -0.263 | -0.1904 | No |
| 5 | PRR11 |  |  | 361 | -0.269 | -0.2342 | No |
| 6 | CDC25B |  |  | 472 | -0.282 | -0.3124 | No |
| 7 | ECT2 |  |  | 481 | -0.283 | -0.3100 | No |
| 8 | TEX30 |  |  | 592 | -0.298 | -0.3877 | No |
| 9 | MCM3 |  |  | 625 | -0.305 | -0.4035 | No |
| 10 | PRC1 |  |  | 645 | -0.310 | -0.4089 | No |
| 11 | DNMT1 |  |  | 659 | -0.312 | -0.4096 | No |
| 12 | CHAF1A |  |  | 679 | -0.315 | -0.4148 | No |
| 13 | NCAPD2 |  |  | 811 | -0.347 | -0.5076 | No |
| 14 | CENPM |  |  | 818 | -0.349 | -0.5015 | No |
| 15 | BUB3 |  |  | 826 | -0.351 | -0.4962 | No |
| 16 | SHCBP1 |  |  | 844 | -0.356 | -0.4986 | No |
| 17 | TCF19 |  |  | 890 | -0.369 | -0.5227 | No |
| 18 | CKAP2 |  |  | 900 | -0.375 | -0.5182 | No |
| 19 | FANCA |  |  | 910 | -0.378 | -0.5136 | No |
| 20 | TUBB4B |  |  | 916 | -0.380 | -0.5057 | No |
| 21 | CDKN2D |  |  | 942 | -0.390 | -0.5134 | No |
| 22 | DIAPH3 |  |  | 943 | -0.390 | -0.5013 | No |
| 23 | SPDL1 |  |  | 995 | -0.412 | -0.5288 | No |
| 24 | LIG1 |  |  | 1035 | -0.437 | -0.5461 | Yes |
| 25 | CEP152 |  |  | 1040 | -0.439 | -0.5356 | Yes |
| 26 | SMC2 |  |  | 1062 | -0.452 | -0.5382 | Yes |
| 27 | EZH2 |  |  | 1070 | -0.455 | -0.5297 | Yes |
| 28 | PLK1 |  |  | 1082 | -0.460 | -0.5241 | Yes |
| 29 | ZWINT |  |  | 1099 | -0.467 | -0.5222 | Yes |
| 30 | RACGAP1 |  |  | 1112 | -0.479 | -0.5169 | Yes |
| 31 | PCNA |  |  | 1142 | -0.504 | -0.5242 | Yes |
| 32 | MXD3 |  |  | 1153 | -0.514 | -0.5161 | Yes |
| 33 | GMNN |  |  | 1155 | -0.516 | -0.5009 | Yes |
| 34 | DBF4 |  |  | 1157 | -0.520 | -0.4856 | Yes |
| 35 | MIS18BP1 |  |  | 1162 | -0.524 | -0.4725 | Yes |
| 36 | CIP2A |  |  | 1165 | -0.533 | -0.4575 | Yes |
| 37 | BRCA1 |  |  | 1171 | -0.541 | -0.4447 | Yes |
| 38 | SGO2 |  |  | 1176 | -0.546 | -0.4309 | Yes |
| 39 | DHFR |  |  | 1197 | -0.576 | -0.4289 | Yes |
| 40 | CKS1B |  |  | 1198 | -0.577 | -0.4110 | Yes |
| 41 | CLSPN |  |  | 1212 | -0.605 | -0.4025 | Yes |
| 42 | CENPK |  |  | 1220 | -0.619 | -0.3888 | Yes |
| 43 | DUT |  |  | 1223 | -0.633 | -0.3708 | Yes |
| 44 | SMC4 |  |  | 1224 | -0.638 | -0.3510 | Yes |
| 45 | CKS2 |  |  | 1226 | -0.641 | -0.3319 | Yes |
| 46 | UBE2T |  |  | 1256 | -0.719 | -0.3325 | Yes |
| 47 | HMGB2 |  |  | 1263 | -0.736 | -0.3144 | Yes |
| 48 | ATAD2 |  |  | 1275 | -0.775 | -0.2991 | Yes |
| 49 | PCLAF |  |  | 1281 | -0.828 | -0.2773 | Yes |
| 50 | HIST2H2AC |  |  | 1288 | -0.880 | -0.2548 | Yes |
| 51 | KPNA2 |  |  | 1297 | -0.972 | -0.2309 | Yes |
| 52 | FBXO5 |  |  | 1301 | -0.992 | -0.2025 | Yes |
| 53 | HIST1H1E |  |  | 1309 | -1.319 | -0.1671 | Yes |
| 54 | HIST1H1C |  |  | 1310 | -1.391 | -0.1240 | Yes |
| 55 | CDK1 |  |  | 1311 | -1.433 | -0.0796 | Yes |
| 56 | HIST1H4C |  |  | 1320 | -2.769 | -0.0000 | Yes |
Table: GSEA details [plain text format]

  

Fig 2: NB\_KI\_CLUSTER5: Random ES distribution      
 Gene set null distribution of ES for **NB\_KI\_CLUSTER5**
